# Formation and Properties of Laser-Induced Periodic Surface Structures on Different Glasses

**DOI:** 10.3390/ma10080933

**Published:** 2017-08-10

**Authors:** Stephan Gräf, Clemens Kunz, Frank A. Müller

**Affiliations:** Otto Schott Institute of Materials Research (OSIM), Friedrich Schiller University Jena, Löbdergraben 32, 07743 Jena, Germany; clemens.kunz@uni-jena.de (C.K.); frank.mueller@uni-jena.de (F.A.M.)

**Keywords:** fs-laser, laser-induced periodic surface structures (LIPSS), fused silica, soda-lime-silicate glass, borosilicate glass, melt formation, viscosity

## Abstract

The formation and properties of laser-induced periodic surface structures (LIPSS) was investigated on different technically relevant glasses including fused silica, borosilicate glass, and soda-lime-silicate glass under irradiation of fs-laser pulses characterized by a pulse duration *τ* = 300 fs and a laser wavelength *λ* = 1025 nm. For this purpose, LIPSS were fabricated in an air environment at normal incidence with different laser peak fluence, pulse number, and repetition frequency. The generated structures were characterized by using optical microscopy, scanning electron microscopy, focused ion beam preparation and Fast-Fourier transformation. The results reveal the formation of LIPSS on all investigated glasses. LIPSS formation on soda-lime-silicate glass is determined by remarkable melt-formation as an intra-pulse effect. Differences between the different glasses concerning the appearing structures, their spatial period and their morphology were discussed based on the non-linear absorption behavior and the temperature-dependent viscosity. The findings facilitate the fabrication of tailored LIPSS-based surface structures on different technically relevant glasses that could be of particular interest for various applications.

## 1. Introduction

The glasses are the material of choice for numerous high-tech applications including e.g., optics, solar cells, microfluidics, and biomaterials. An important aspect of these glasses is related to their surface-specific properties including absorption, reflection, and wettability that can be tailored by engineering the surface. Ultra-short pulsed lasers (fs-lasers) demonstrated to be a versatile tool for the fabrication of functional structures inside the bulk of transparent materials (e.g., nanogratings) [1,2], as well as on the surface of materials [3]. In the context of surface structuring, laser-induced periodic surface structures (LIPSS) gained rapidly increasing attention over the last decade [4,5], although their first observation by Birnbaum dates back to the year 1965 [6]. According to the spatial period, LIPSS are typically classified into low-spatial frequency LIPSS (LSFL) and high-spatial frequency LIPSS (HSFL). On dielectrics, LSFL often show spatial periods close to the utilized laser wavelength *λ* or close to *λ*/*n*, with *n* being the refractive index of the dielectric material. The orientation of the LSFL is correlated to the laser beam polarization, whereas the alignment on dielectrics is either perpendicular or for large band gap materials (e.g., SiO_2_, BaF_2_) parallel to the electrical field vector [7]. In the 1980’s, several research groups provided theories for the formation of LSFL [8,9,10]. It is generally accepted that their formation mechanism is related to a spatially modulated energy deposition pattern resulting from the interference of the incident laser radiation with excited surface electromagnetic waves, which may involve the excitation of surface plasmon polaritons [8]. HSFL with periods much smaller than *λ* are predominantly observed for the irradiation with pulses in the ps- to fs-range mainly for below band-gap excitation of transparent materials [7,11]. Their origin, however, still remains unclear. Hence, numerous investigations are still under research. Possible explanations include self-organization [12], second-harmonic generation [13], and chemical surface alterations [14].

The formation of LIPSS on fused silica has already been studied considering several influencing parameters including the laser peak fluence, *F*, the number of incident laser pulses, *N*, and the beam polarization mainly for the irradiation with Ti:sapphire fs-laser pulses (*λ* = 800 nm) in an air atmosphere [7,15,16,17,18,19,20,21,22,23]. However, the chemical composition of a glass significantly determines its physical properties including the glass transition from the solid and the liquid state, which is accompanied by a change of the viscosity over several orders of magnitude. Consequently, the interaction between laser radiation and matter, the LIPSS formation process and its corresponding threshold fluences, as well as the properties and morphologies of the fabricated LIPSS are strongly determined by the specific composition of the glass. In the present paper, the formation of LIPSS on fused silica, borosilicate glass, and soda-lime-silicate glass was systematically investigated using fs-laser radiation with different laser peak fluence, pulse number, and repetition frequency.

## 2. Results and Discussion

### 2.1. LIPSS Formation on Different Glasses

**Figure 1:**
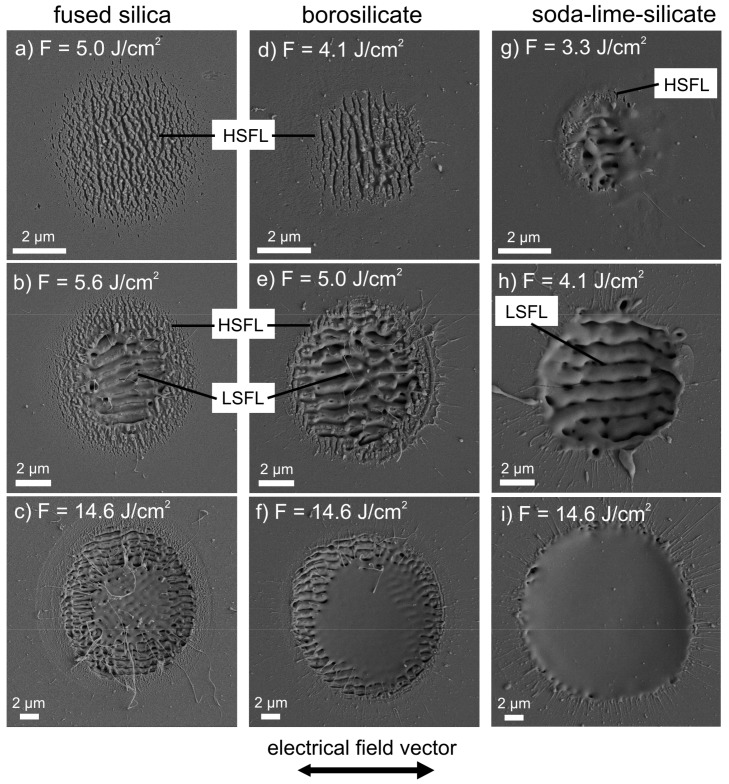
SEM micrographs of the surface of fused silica (**a**–**c**), borosilicate glass (**d**–**f**), and soda-lime-silicate glass (**g**–**i**) upon irradiation with *N* = 5 linearly polarized laser pulses of different peak fluences, *F*, at a repetition frequency *f*_rep_ = 1 kHz. Please note the direction of the electrical field vector and the different scaling of the micrographs, which was chosen in order to display the entire ablation spot.

Figure 1 shows SEM micrographs of the surface of fused silica (Figure 1a–c), borosilicate glass (Figure 1d–f), and soda-lime-silicate glass (Figure 1g–i) after irradiation with *N* = 5 linearly polarized laser pulses of different fs-laser peak fluence, *F*, between 3.3 J/cm^2^ and 14.6 J/cm^2^ using a repetition frequency *f*_rep_ = 1 kHz. The specific values of *F* used for the different glasses were chosen with respect to the different threshold values *F*_th_^LSFL^ for LSFL formation (see Section 2.2), i.e., *F* was set close to *F*_th_^LSFL^ to verify HSFL formation and larger than *F*_th_^LSFL^ to receive a well-pronounced LSFL pattern. The fluence value *F* = 14.6 J/cm^2^, which corresponds to the maximum peak fluence of the fs-laser, was utilized to investigate melt formation.

From Figure 1 it becomes evident that generally both types of LIPSS, HSFL, and LSFL, can be found on all types of investigated glasses. In the case of fused silica, the lowest fluence value, *F* = 5.0 J/cm^2^ leads to the formation of HSFL (Figure 1a) with an orientation perpendicular to the direction of the electrical field (E-field) vector of the fs-laser radiation. The increase of *F* to 5.6 J/cm^2^ results in the formation of LSFL in the intense center of the Gaussian beam profile, which are surrounded by a ring-shaped area containing well-pronounced HSFL (Figure 1b). A further increase of *F* to 14.6 J/cm^2^ increases the diameter *D*_LSFL_ of the area covered with LSFL and finally results in a homogenous pattern of LSFL. It is aligned parallel to the beam polarization and surrounded by HSFL (Figure 1c). The corresponding SEM micrograph reveals a small area centered in the ablation spot where melt formation occurred. Beyond, filaments of solidified fused silica can be observed that are arranged over the entire ablation spot in radial direction. The results obtained for fused silica are in good agreement with literature results [18].

LIPSS formation on borosilicate glass (Figure 1d–f) and soda-lime-silicate glass (Figure 1g–i) occurs in a way equal to fused silica and leads, at a first glance, to similar morphology and alignment of the LIPSS pattern. Nevertheless, the LIPSS formation occurs in different fluence ranges due to the different ablation thresholds (see Section 2.2). Beyond, main differences between the investigated glasses are related to the following observations:

(1) The size of the ring-shaped area with HSFL that surrounds the LSFL in the intense center is smaller in the case of borosilicate glass (Figure 1e) and cannot be detected for soda-lime-silicate glass in the investigated fluence range (Figure 1h).
(2) LIPSS formation on soda-lime-silicate glass is remarkably determined by melt formation in the entire investigated fluence range. Even at the lowest fluence, *F* = 3.3 J/cm^2^ (Figure 1g), HSFL are only barely visible. Although the SEM micrograph shows certain points with HSFL-like structures, their formation and appearance is difficult to verify. Moreover, due to melt formation the morphology of the homogenous LSFL pattern fabricated with *F* = 4.1 J/cm^2^ (Figure 1h) differs remarkably from LSFL on fused silica and borosilicate glass (Figure 1b,e) generated with the corresponding fluences required for LSFL formation (*F* > *F*_th_^LSFL^).
(3) At the highest value *F* = 14.6 J/cm^2^, the area where melt formation occurs is strongly increased on borosilicate glass (Figure 1f) when compared to fused silica and covers the total ablation spot for soda-lime-silicate glass (Figure 1i). In both cases, the surface of this centered area is very flat without any LIPSS-like surface modulation.

**Figure 2:**
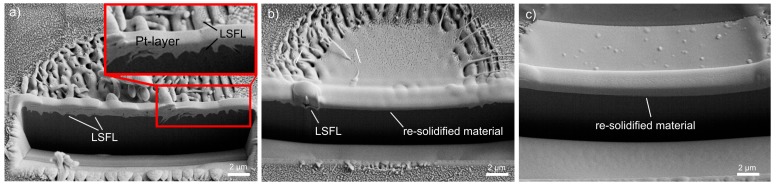
FIB cross-sections of the ablation spots containing low-spatial frequency LIPSS (LSFL) fabricated with *F* = 14.6 J/cm^2^ on (**a**) fused silica, (**b**) borosilicate glass, and (**c**) soda-lime-silicate glass.

These areas were analyzed in detail by preparing FIB cross-sections. Figure 2 shows that LSFL can be observed over almost the entire ablation spot in the case of fused silica (Figure 2a). On the contrary, borosilicate glass (Figure 2b) and soda-lime-silicate glass (Figure 2c) exhibit very flat surface profiles. Moreover, possible sub-surface LIPSS structures that might be covered with a thin melt-layer can be excluded. The results indicate an intensive heating in the center of the ablation spot leading to melt formation. Moreover, this increase in temperature causes a reduction of the temperature-dependent viscosity, which will be discussed in detail in Section 2.3.

### 2.2. LSFL Formation Threshold

**Figure 3:**
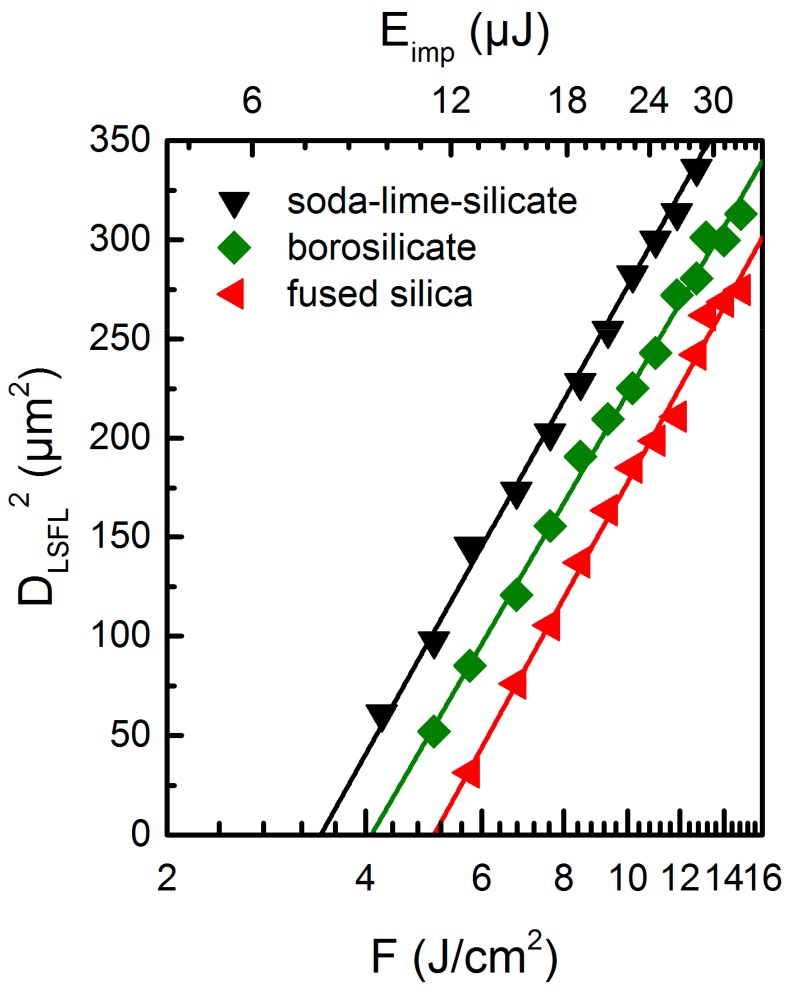
Evaluation of the threshold fluence required for the formation of LSFL on different glasses using *N* = 5 linearly polarized fs-laser pulses and a repetition frequency *f*_rep_ = 1 kHz according to the method proposed by Liu [24].

The SEM micrographs in Figure 1 indicate a transition from HSFL to LSFL at a specific threshold fluence *F*_th_^LSFL^, which was quantified by plotting *D*^2^_LSFL_ in a semilog plot versus *F* as proposed by Liu [24] (Figure 3). Extrapolating the linear fit of the measured diameters to zero provides the values of *F*_th_^LSFL^ of the investigated glasses, which were found to be 5.1 J/cm^2^ for fused silica, 4.1 J/cm^2^ for borosilicate, and 3.4 J/cm^2^ for soda-lime-silicate glass, respectively, considering the used processing conditions. These threshold values are in good agreement with the observations obtained from the SEM micrographs.

The formation of LSFL is based on the excitation, heating, and the selective ablation of the materials surface. The corresponding threshold fluence is determined by the energy density required to dissociate the oxidic composition of the glass into its atomic components and by the absorption behavior of the material, which is based on non-linear multiphoton processes [25]. The absorption of the material strongly depends on the band gap energy *E*_g_, which is about 9 eV for fused silica [26]. Using transmission spectroscopy, *E*_g_ of soda-lime-silicate glass and borosilicate glass was determined to be 3.9 eV and 4.4 eV, respectively, in line with literature values [27,28]. The utilized laser wavelength *λ* = 1025 nm corresponds to an energy of a single photon *E*_ph_ ≈ 1.2 eV. Consequently, the absorption of the laser radiation occurs via the excitation of electrons over the band gap into the conduction band by non-linear multi-photon processes. These quasi-free electrons are able to absorb further photons in a linear absorption process. Once the energy of these electrons exceeds *E*_g_, it can be transferred to valence band electrons, which then are excited into the conduction band. This process is referred to as impact or avalanche ionization. Considering the different band gap energies, a multiphoton absorption requires at least four photons in the case of borosilicate and soda-lime-silicate glass and eight photons for fused silica to fulfill the condition *m*∙*E*_ph_ ≥ *E*_g_ for multi-photon absorption (*m*: number of photons). To discuss the vaporization process of a glass, its specific chemical composition needs to be addressed. All investigated glasses belong to the group of silica (SiO_2_) based glasses with different concentrations of network modifying oxides (e.g., Na_2_O, K_2_O) and network forming oxides (e.g., B_2_O_3_). Such oxides strongly determine the physical properties of the glasses e.g., its viscosity. As described by Sun and Huggins, the average dissociation energy per unit volume of a multicomponent glass can be calculated when taking into account the dissociation energies of the involved oxides, which have to be weighted with their corresponding molar fraction [29,30]. With the dissociation energies of the glass oxides taken from Grehn et al. [25], the average dissociation energies of the investigated glasses were calculated to be 62.6 kJ/cm^3^ for soda-lime-silicate glass, 63.2 kJ/cm^3^ for borosilicate glass, and 64.9 kJ/cm^3^ for fused silica. Taking into account the specific number of photons required to excite an electron into the conduction band in the different glasses, these calculated dissociation energies can be used to explain the measured threshold fluence that increases from soda-lime-silicate glass via borosilicate glass to fused silica.

### 2.3. Melt Formation and Viscosity

The SEM micrographs in Figure 1 reveal differences in the melt formation process during the fabrication of LIPSS for the investigated glasses. For soda-lime-silicate glass, it leads to a remarkable modification of the LSFL morphology even at the lowest F. For the highest laser peak fluence, the ablation crater is completely covered with a melt layer, i.e., LSFL cannot be observed. In order to identify the reasons of melt formation, the interaction process was studied for the different glasses in dependence on the pulse number.

**Figure 4:**
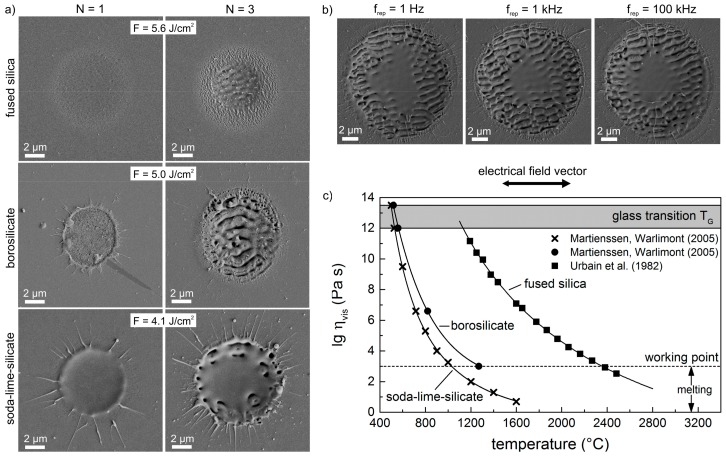
(**a**) SEM micrographs of single spots on the surface of fused silica, borosilicate glass, and soda-lime-silicate glass upon irradiation with a single pulse (*N* = 1) and *N* = 3 pulses of different laser peak fluence *F*; (**b**) SEM micrographs of LSFL on borosilicate glass upon irradiation with *N* = 5 linearly polarized laser pulses using a peak fluence *F* = 8.3 J/cm^2^ and different repetition frequencies between 1 Hz and 100 kHz; (**c**) Viscosity *η*_vis_ of the investigated glasses in dependence on temperature [31,32].

Figure 4a shows SEM micrographs of the investigated glasses upon irradiation with a single pulse (*N* = 1) und *N* = 3 laser pulses, respectively. The utilized fluences *F* = 4.1 J/cm^2^, 5.0 J/cm^2^, and 5.6 J/cm^2^ correspond to the laser fluences that are required for the fabrication of homogenous LSFL pattern in the entire ablation spot as shown in Figure 1. It becomes evident for fused silica, that the surface exhibits a slightly increased roughness after irradiation with a single pulse. Subsequently, in this modified area the irradiation of two further pulses (*N* = 3) leads to the formation of HSFL in the less-intensive region and LSFL in the intense center of the Gaussian laser spot. In comparison to the final LSFL pattern (Figure 1b), the appearing LSFL are still less pronounced. After the single pulse interaction, the surface of borosilicate glass also exhibits a slightly roughened surface in the focal spot with some isolated pores, which indicate a gentle melting and vaporization of the surface.

In direct vicinity to this area, a ring with a diameter of about 5 µm consisting of molten and re-solidified material can be observed, whose origin has already been described for borosilicate glasses by Ben-Yakar and co-workers [33], who state that thermocapillary forces (Marangoni flow) and forces by the pressure of the plasma above the surface exert on this melt layer and drive the liquid from the center to the edges of the crater to create a rim as the melt re-solidifies. After three pulses (*N* = 3), the LSFL are clearly pronounced with a narrow ring containing HSFL. Upon single pulse irradiation of soda-lime-silicate glass, a circular molten area with a diameter of about 5 µm can be obtained at the surface, that evolves to a significant amount of molten material after two further pulses (*N* = 3). Although LSFL can only be observed in parts at this state, a narrow fluence range still leads to the formation of a homogenous LSFL pattern after the irradiation of *N* = 5 pulses (Figure 1h). Pulse number dependent investigations demonstrate that melt formation occurs on soda-lime-silicate and borosilicate glass already after the irradiation of a single pulse even at moderate laser fluences. Therefore, it can be related to intra-pulse effects. However, additional inter-pulse effects such as heat accumulation due to the sequential irradiation of laser pulses might also contribute to melt formation. Therefore, LIPSS formation was investigated upon irradiation with *N* = 5 linearly polarized pulses at different repetition frequencies *f*_rep_ between 1 Hz und 100 kHz (Figure 4b). The time interval between two successive pulses was consequently varied from 1 s down to 10 µs, which provides different time durations for the material to cool down. The experiments were conducted on borosilicate glass with *F* = 8.3 J/cm^2^, which ensures moderate melt formation in the center of the spot. It becomes evident from the SEM micrographs in Figure 4b that both the diameter of the ablation crater and the morphology of the fabricated LSFL are independent on the repetition frequency. Moreover, the size of the molten and re-solidified flat centered area kept unaffected by *f*_rep_, whereas heat accumulation effects can be excluded.

The results shown in Figure 4 can be explained by the temperature-dependency of the viscosity *η*_vis_. Generally, glass is considered to be a frozen, super-cooled liquid, for which no sharp melting point can be defined. The transition from the solid to liquid state is rather characterized by a temperature range, the so-called glass transition *T*_g_. Here, the glass has a very high viscosity that decreases rapidly with increasing temperature (Figure 4c). It is shown that *T*_g_ of borosilicate glass and soda-lime-silicate glass is in the order of about 600 °C, while *T*_g_ of fused silica is almost twice as high. Furthermore, the investigated glasses exhibit strongly varying dependencies of the viscosity on temperature. By definition, a viscosity of *η*_vis_ = 10^3^ Pa∙s corresponds to the working point, where the glass is easily deformed. The melting point of the glass is defined at *η*_vis_ = 10^1^ Pa∙s. However, fused silica reaches the defined melting point at *T* ≈ 3000 °C, which is almost identical with the vaporization temperature [34]. This means in fact that the molten fused silica surface is characterized by a relatively high *η*_vis_ even just below vaporization temperature. For comparison, an iron melt exhibits a much lower viscosity of *η*_vis_ = 4 × 10^−4^ Pa∙s already at melting temperature [35]. As discussed for the ablation threshold, the vaporization temperature of the multi-component glasses (soda-lime-silicate and borosilicate glass) is very difficult to define due to the vaporization of the different oxides. Nevertheless, the melting points (*η*_vis_ = 10^1^ Pa∙s) of soda-lime-silicate and borosilicate glass are already reached at around 1500 °C and 1800 °C, respectively. Consequently, both molten glasses attain remarkably lower viscosities during further increasing temperature and the range of their molten state between *T*_g_ and the complete vaporization of the liquid is much wider. This means that the melt exhibits a higher fluidity and that the active forces (thermocapillary forces, plasma pressure) gained more time to influence the melt and LSFL formation process, as it was observed in the SEM micrographs. Finally, the lower viscosity of soda-lime-silicate and borosilicate glass when compared to fused silica might explain the remarkable reduction of the ring-shaped area containing HSFL. It is expected that the low viscosity of the softened surface leads to a closure of the very fine surface structures. Thus, HSFL cannot be observed.

### 2.4. Theoretical Analysis of LIPSS Formation

**Figure 5:**
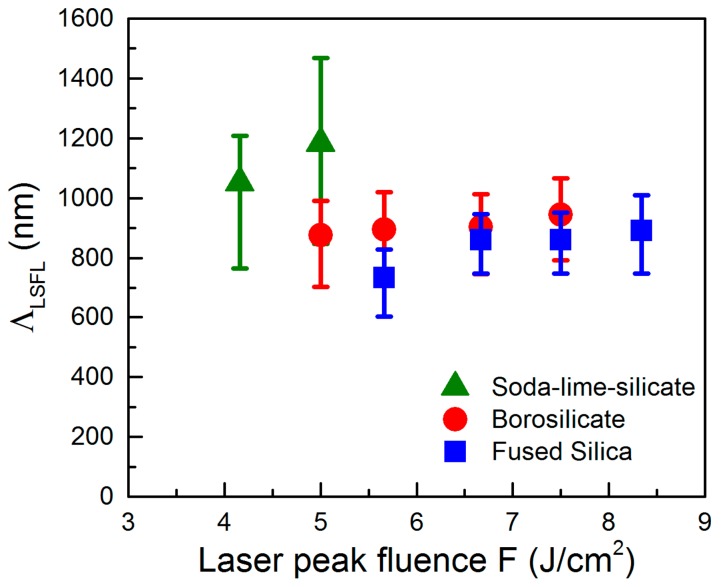
Spatial periods Λ_LSFL_ of the LSFL on different glasses in dependence on the laser peak fluence, F. The fluence ranges provide homogenous LSFL pattern without melt formation. The error bars indicate the width of distribution of the corresponding FFT operation.

Figure 5 shows the spatial periods Λ_LFSL_ of LSFL obtained from Fast-Fourier transform (FFT) of the corresponding SEM micrographs for different glasses. The analyses consider only the fluence ranges that provide homogenous LSFL pattern without melt formation. Taking into account the width of the FFT operation, which is illustrated by the error bars in Figure 5, LSFL with spatial periods between 600 nm and 1007 nm (0.59 × *λ*–0.98 × *λ*) can be observed on the surface of fused silica. With increasing peak fluence F, the corresponding center positions of the characteristic peaks of the FFT operations slightly increase from 735 nm to 890 nm. In the case of borosilicate glass, a moderate fluence-dependent increase of the center positions from 877 nm to 950 nm can be observed. It becomes evident, that Λ_LSFL_ is larger at the respective fluence values when compared to fused silica. The width of the FFT operation leads to spatial periods ranging from 703 nm to 1067 nm (0.69 × *λ*–1.04 × *λ*).

Figure 5 shows that the formation of LSFL on soda-lime-silicate glass is limited to a very narrow fluence window because of the observed strong melt formation. Here, the determined center positions of the FFT peaks are 1052 nm for *F* = 4.1 J/cm^2^ and 1184 nm for *F* = 5.0 J/cm^2^, i.e., they exceed the initial laser wavelength *λ*. Beyond, they are remarkably larger as those found on borosilicate glass and fused silica. Moreover, the error bars reveal a larger width of distribution of the FFT, i.e., a wider range of the spatial periods, which was determined to reach from 764 nm to 1466 nm (0.75 × *λ*–1.43 × *λ*).

The theoretical analysis of LIPSS formation and the corresponding spatial periods bases on the theory of Sipe et al. [8], which was discussed in detail for fused silica and other materials by Bonse, Höhm and co-workers [18,36,37]. Briefly, this theory predicts potential LIPSS wave vectors ***k*** of the surface (**k** = 2π/*λ*) as a function of the laser parameters (wavelength *λ*, angle of incidence *Θ*, polarization direction) and materials properties (dielectric constant *ε*, surface roughness). As a result, it introduces the so-called efficacy factor *η* that determines the efficacy of a surface to absorb energy at the wave vector **k**, i.e., the theory facilitates the prediction of spatial periods of LIPSS. The optical properties of non-excited materials are determined by a complex dielectric function *ε* = *ε*_r_ + i*ε*_i_, where the optical constants (*n*: refractive index, *k*: extinction coefficient) are integrated via *ε* = n*^2^ = (*n* + i*k*)^2^. For the investigated glasses at *λ* = 1025 nm, the extinction coefficient *k* can be neglected, i.e., *ε* is given by *ε* = *n*^2^. The transient change of the optical properties due to the excitation of quasi-free electrons can be described by a Drude model, which provides the dielectric function ε* of the laser-excited material by adding the additional Drude term Δ*ε_D_* to *ε*:

(1) $$\varepsilon^{*}=\varepsilon +\Delta \varepsilon_{D}=\varepsilon -\frac{e^{2}\cdot N_{e}}{\varepsilon_{0}\cdot m_{opt}\cdot m_{e}\cdot \omega^{2}}\left(1+\frac{i}{\omega \cdot \tau_{D}}\right)$$

Here, *e* represents the electron charge, *N*_e_ is the laser-induced electron density, *m*_e_ is the electron mass, *ε*_0_ is the vacuum dielectric permittivity and *ω* the laser angular frequency. For the calculations we used a Drude damping time of *τ*_D_ = 0.4 fs and an optical effective mass of *m*_opt_ = 0.49, which demonstrated to be suitable parameters for fused silica [38]. Specific values for borosilicate glass and soda-lime-silicate glass are missing in literature. However, it becomes evident, that the efficacy factor calculation is only insignificantly affected by a moderate variation of the Drude parameters. The above-mentioned values for fused silica are therefore used for the calculation for all investigated glasses. The shape factor, *s* and the filling factor, *f* were set to 0.4 and 0.1, respectively [8].

**Figure 6:**
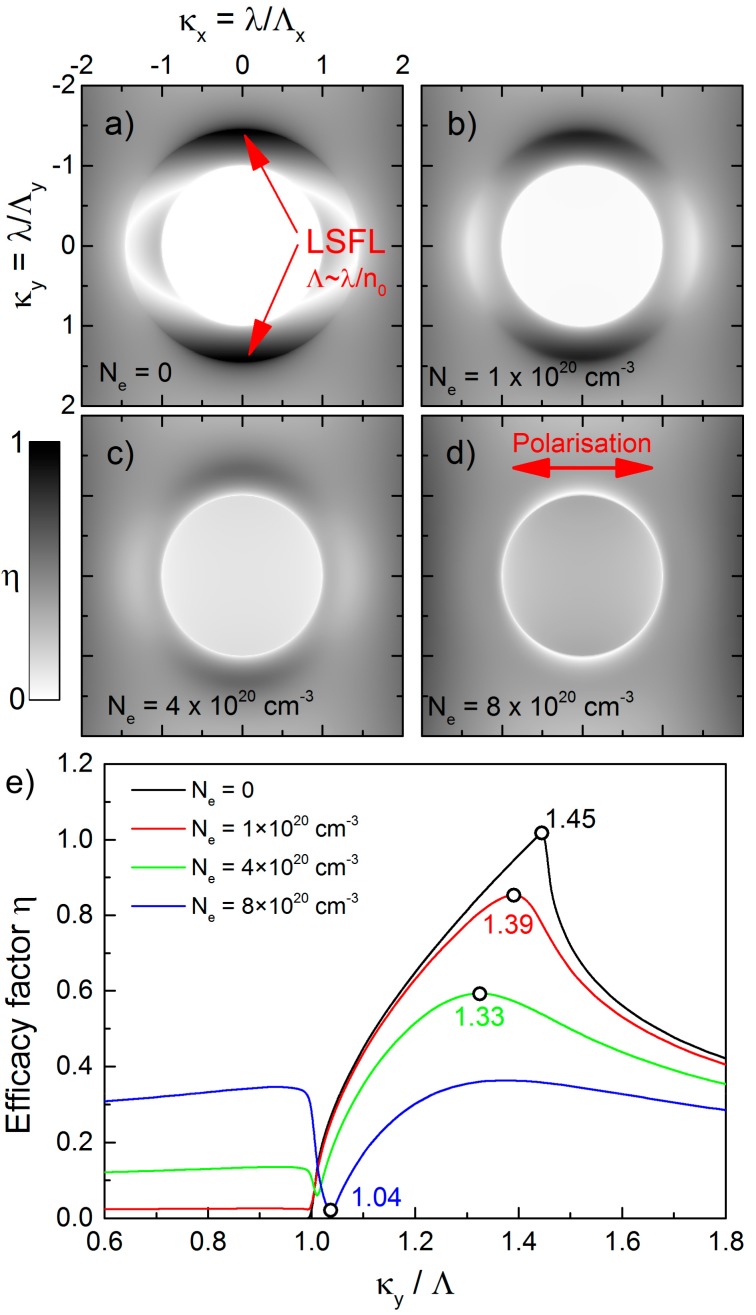
Efficacy factor, *η*, calculated for fused silica as a function of the normalized LIPSS wave vectors *κ*_x_, *κ*_y_ with *λ* = 1025 nm and *Θ* = 0°: (**a**–**d**) Two-dimensional grey-scale images of *η* and e) cross-sections of the *η*-maps along the positive *κ*_y_-direction at *κ*_x_ = 0 in dependence on the quasi-free electron density *N*_e_ [(**a**) *N*_e_ = 0 (*n* = *n*_0_ = 1.4504); (**b**) *N*_e_ = 1 × 10^20^ cm^−3^ (*n* = 1.4273, *k* = 0.032); (**c**) *N*_e_ = 4 × 10^20^ cm^−3^ (*n* = 1.3609, *k* = 0.1347); (**d**) *N*_e_ = 8 × 10^20^ cm^−3^ (*n* = 1.2829, *k* = 0.2858)]. The circles mark the *κ*_y_-position where the formation of LSFL is expected [8].

Figure 6a–d shows the resulting two-dimensional grey-scale images of *η* calculated for fused silica as a function of the normalized LIPSS wave vector components *κ*_x_, *κ*_y_ (|*κ*| = *λ*/*Λ*) in dependence on *N*_e_. For the non-excited material (*N*_e_ = 0, *ε* = ε* = 2.1036), the grey-scale image (Figure 6a) exhibits arc-like shaped regions, where the efficacy factor is increased. Sharp *η*-maxima at *λ*/*Λ*_y_ = ±1.45 can be obtained, which correspond to the refractive index *n*_0_ of non-excited fused silica (*Λ* ~ *λ*/*n*) [39]. It can be associated to LSFL with spatial periods around *Λ* ~ *λ*/*n*_0_ = 707 nm and an orientation parallel to the beam polarization. The deviation of the experimental spatial period *Λ*_LSFL_ = (735 ± 93) nm observed at the lowest fluence (*F* = 5.6 J/cm^2^) can be explained by the fact that the theory of Sipe refers to a single-pulse interaction process. Therefore, inter-pulse effects such as incubation and grating-assisted coupling are not considered although they strongly influence the formation process [40,41]. Beyond, the modification of the optical properties caused by the intense laser irradiation has to be considered. Figure 5b–d illustrates that the increase of *N*_e_ leads to a modification of the arc-like shaped regions, which also corresponds to the cross-sections of the *η*-maps along the positive *κ*_y_-direction at *κ*_x_ = 0 (Figure 6e). According to Sipe et al., LIPSS can be expected where *η* exhibits a sharp maxima or minima [8]. The graphs calculated for different values of *N*_e_ demonstrate that with increasing *N*_e_ the position of the *η*-maximum is shifted towards smaller *κ*_y_-values, i.e., in the direction of larger spatial periods. Simultaneously, the contour of the arc-like shaped regions starts to widen, which might explain the relatively wide distribution of the measured spatial periods represented by the error bars. The shift of the LSFL features can be used to explain the slight increase of the spatial periods of the individual glasses in dependence on the laser peak fluence *F* (Figure 5). With increasing *F*, more electrons are excited into the conduction band. This results in an increase of *N*_e_ and consequently of *Λ*_LSFL_. From the similar refractive indices (fused silica: *n*_0_ = 1.4504, borosilicate glass: *n*_0_ = 1.4626, soda-lime-silicate glass: *n*_0_ = 1.5134) [39,42,43] one could expect that the spatial periods *Λ*_LSFL_ only slightly differ for the investigated glasses following the above discussed dependency *Λ* ~ *λ*/*n* for the non-excited material (Figure 6a). This is in contrast to the experimental results shown in Figure 5a, which might be explained by the different band gap energies. As a result, the glasses are subjected to a varying degree of excitation, which increases from fused silica via borosilicate glass to soda-lime-silicate glass. Consequently, an increasing number of excited electrons leads to larger spatial periods *Λ*_LSFL_. However, it has to be noted that the relatively high *Λ*_LSFL_-values obtained for soda-lime-silicate glass, which exceed the initial laser wavelength *λ*, cannot be predicted by the efficacy factor theory. Figure 6e demonstrates that the position of the *η*-maximum is shifted from the initial value of the non-excited material *κ*_y_/*Λ* = 1.45 towards *κ*_y_/*Λ* ≈ 1. However, values of *κ*_y_/*Λ* < 1, which would be required to explain the observed central peaks of the FFT operation (e.g., *κ*_y_/*Λ* = 0.86 for *Λ*_LSFL_ = 1184 nm), are not achieved. On the contrary, Höhm et al., reported that strongly excited fused silica exhibits a metal-like behavior that leads to LSFL with *Λ*_LSFL_ ≈ *λ* accompanied by an alignment perpendicular to the polarization direction [13]. However, these structures were not detected by the authors in their experiments. In our opinion, it has to be considered that LIPSS-formation on soda-lime-silicate glass is remarkably determined by melt formation that already occurs during the interaction of the very first fs-laser pulse with the material (Figure 4a). Therefore, hydrodynamic processes and instabilities within the melt phase might lead to a modification of the formation process and the resulting morphology of the LSFL [34,44,45]. This is less pronounced for borosilicate glass when compared to soda-lime-silicate glass because of a lower degree of melt formation and a larger viscosity.

## 3. Materials and Methods 

LIPSS were generated using a diode pumped Yb:KYW thin disc fs-laser system (JenLas D2.fs, Jenoptik, Jena, Germany) as the radiation source. The emitted linearly polarized laser pulses are characterized by a pulse duration *τ* = 300 fs, a central wavelength *λ* = 1025 nm, and pulse energies *E*_imp_ up to 40 µJ. The pulsed laser beam was focused by a galvanometer scanner (IntelliScan14, Scanlab, Puchheim, Germany) including a f-Theta objective (JENar, Jenoptik, Jena, Germany) with a focal length *f*_L_ = 100 mm. Using the method of Liu [24], the resulting focal spot diameter was measured to 2*w*_f_ = (24 ± 0.5) µm. The uncertainty of the laser peak fluences *F* = 2 × *E*_imp_/(π × *w*_f_^2^) determined from the measured pulse energy *E*_imp_ are estimated <10%. The investigated glasses were provided by Schott AG Germany and their exact composition was summarized in literature [25,46]. The glass samples were ultrasonically cleaned in acetone and isopropanol before and after laser processing. LIPSS were generated by irradiating the sample surface at normal incidence under ambient air atmosphere. Different values of the fs-laser peak fluence *F*, the pulse number *N* and the repetition frequency *f*_rep_ were chosen to investigate the influence of these parameters on the LIPSS formation on different glasses. Laser processed sample surfaces were subsequently characterized by scanning electron microscopy (SEM). For this purpose, the glass samples were sputtered with gold and examined in the SEM (Sigma VP, Zeiss, Jena, Germany) at an accelerating voltage of 1–5 kV using a secondary electron detector. The spatial periods of the LIPSS pattern were quantified by Fast-Fourier transform (FFT) of the SEM micrographs. In this context, error bars displayed in the graphs indicate the width of distribution of the corresponding FFT operation. Moreover, the ablation diameters were evaluated by optical microscopy (OM). The microscope (VHX-100K, Keyence, Osaka, Japan) was equipped with a wide-range zoom lens (VH-Z500, Keyence, Osaka, Japan) providing a magnification range between 500× and 5000×. Cross-sections of the ablations spots were prepared by using a focused ion beam (FIB) (Helios Nanolab 600i, FEI, Eindhoven, The Netherlands) after a protective platinum layer was deposited on the surface of the sample. The band gap energies of soda-lime-silicate and borosilicate glass were determined by transmission spectroscopy (Tidas MCS/100-3, J&M, Aalen, Germany).

## 4. Conclusions

Laser-induced periodic surface structures have been prepared on fused silica, soda-lime-silicate glass, and borosilicate glass by using fs-laser pulses. In contrast to fused silica and borosilicate glass, soda-lime-silicate glass exhibits remarkable melt-formation, which was revealed as an intra-pulse effect. Moreover, HSFL were not observed and the LSFL are characterized by larger spatial periods. The results can be explained by differences in the non-linear absorption behavior and viscosity of the investigated glasses. Our findings will facilitate the tailored fabrication of LIPSS-based surface structures on different technical relevant glasses and are of particular interest for sophisticated applications in plasmonics [47], microfluidic devices [48], control of cell behavior through patterned surfaces [49,50], and the engineering of surfaces with specific wetting properties [51,52].
